# The meaning of blood pressure

**DOI:** 10.1186/s13054-018-2171-1

**Published:** 2018-10-11

**Authors:** S. Magder

**Affiliations:** Department of Critical Care, McGill University Health Centre, 1001 Decarie Blvd., Montreal, Quebec H4A 3J1 Canada

**Keywords:** Arterial resistance, Conductance, Critical closing pressure, Cardiac output, Blood flow, Perfusion, Gravitational energy, Elastic energy, Kinetic energy

## Abstract

Measurement of arterial pressure is one of the most basic elements of patient management. Arterial pressure is determined by the volume ejected by the heart into the arteries, the elastance of the walls of the arteries, and the rate at which the blood flows out of the arteries. This review will discuss the three forces that determine the pressure in a vessel: elastic, kinetic, and gravitational energy. Emphasis will be placed on the importance of the distribution of arterial resistances, the elastance of the walls of the large vessels, and critical closing pressures in small arteries and arterioles. Regulation of arterial pressure occurs through changes in cardiac output and changes in vascular resistance, but these two controlled variables can sometimes be in conflict.

## Background

Blood pressure is one of the most commonly measured clinical parameters and blood pressure values are major determinants of therapeutic decisions. However, interpretation of the physiological meaning of blood pressure in an individual patient is not always an easy task. This paper reviews the physical basis and physiological determinants of arterial pressure, and the relationship of arterial pressure to tissue perfusion. Some of the issues have been covered in a previous review on blood pressure [1]. The objective of this paper is to provide guidance when considering therapeutic options but it is not possible to give a definitive algorithm with current knowledge.

## Physical basis of vascular pressures

Pressure is a force distributed over a surface area and, as such, it has the same units as tension. The term pressure is used instead of tension because tension is determined in a single direction, whereas pressure can be used over the curved surfaces of vessels and has the units of force per cross-sectional area. Force is the product of mass and acceleration, and the standard unit is a Pascal, which is a newton per square meter. However, vascular pressures still most often are measured in length-based units of millimeters of mercury or centimeters of water. This has historical origins. Before the availability of electronic transducers, pressures were measured with columns of water or mercury. The mass of the column is the product of the volume and density. The density of water is 1 and that of mercury is 13.6 times that of water. The height of a column of fluid is proportional to the volume over the cross-sectional area of the column, and thus has units of length. Pressure, therefore, is proportional to the product of the density of the fluid and the height of the fluid and gives force per cross-sectional area. The force on the column of water, or mercury, is the acceleration of the column by gravity. These “length” measurements of force are relative, for they depend upon the position on the earth relative to the center of the earth. However, the acceleration due to gravity is similar over all the earth’s earth. It is even only about 0.2% lower at the top of Mt. Everest. Thus, length-based units are still useful for biological measurements. Units of millimeters of mercury (mmHg) are converted to kilopascals by multiplying by 0.13.

### Elastic energy

**Fig. 1:**
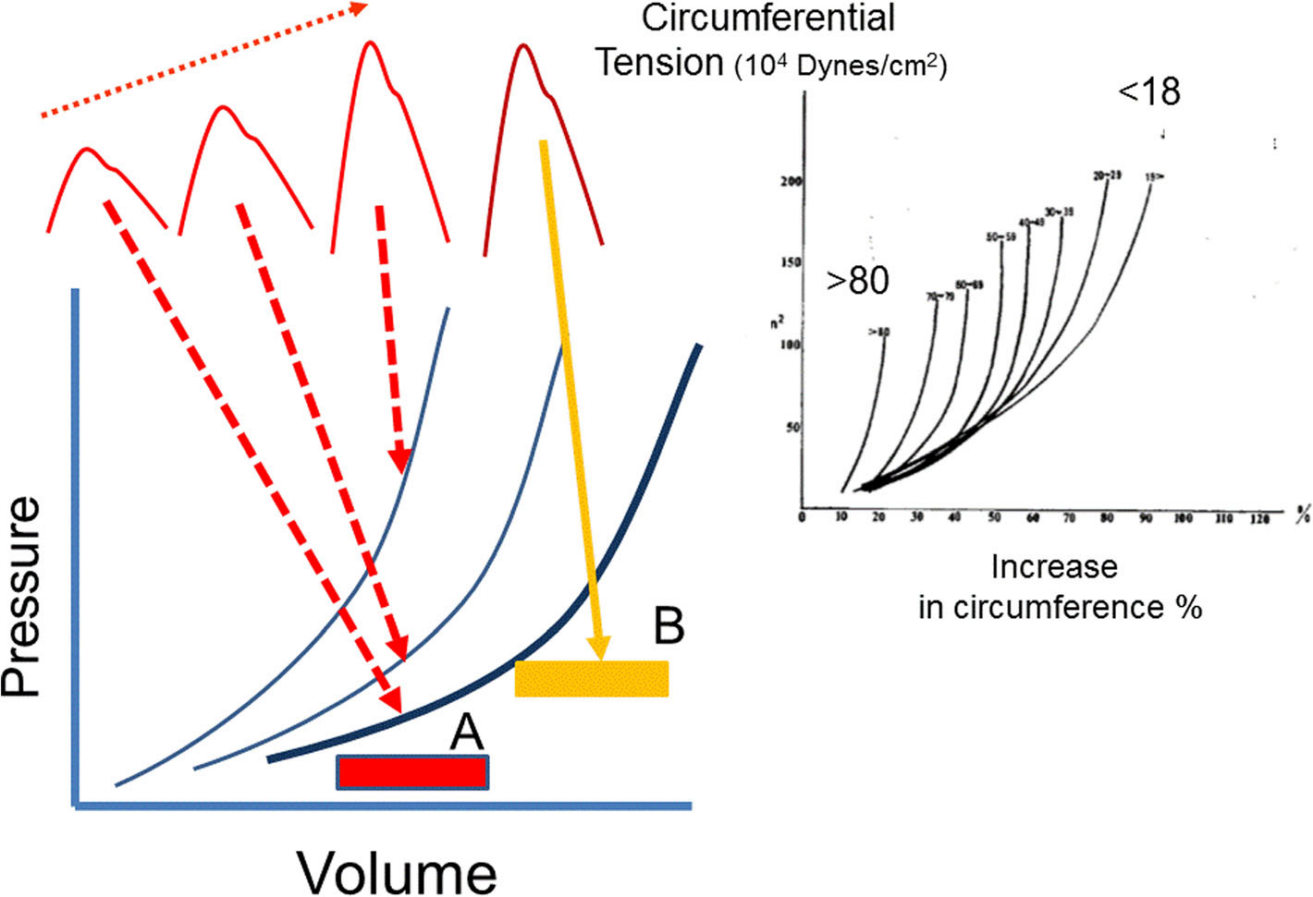
Effect of age and initial volume on thoracic aortic elastance. The slopes of the lines are elastance. The *right upper insert* shows the increase in circumferential tension versus increases in aortic circumference in percent for age < 18 to > 80 years [33]. The *lower left* shows a schematic pressure–volume relationship for the aorta. The *boxes* represent stroke volumes. The same stroke volume *A* starting from the same initial volume produces increasing pulse pressures depending upon the shape and position of the start of the stroke volume. The stroke volume *B* is the same size as in *A* but starts at a higher initial volume and produces a much larger pulse pressure

Three types of energy produce arterial pressure: elastic, kinetic, and gravitational. By far the most significant is elastic energy. The volume inside vascular structures stretches their elastic walls and produces a recoil force, which, based on the elastic properties of the structure, creates a pressure. The materials making up vascular structures are not homogenous so that the volume-to-pressure relationship of arterial vessels is not linear and has a convex curvilinearity [2–4] (Fig. 1). Resistance to stretch of a substance is called elastance and the inverse, the ease of stretch, compliance. Normal blood flow is pulsatile because of the cyclic nature of cardiac emptying and filling. The consequent cyclic changes in the volume of the aorta produce the cyclic changes in arterial pressure. Although the elastance of the wall of arterial vessels varies with volume, over short periods of time the actual curvilinear relationship of volume to pressure is constant because it is determined by the composition of the vascular wall [2, 4]. Changes in this curvilinear relationship of aortic elastance require changes in the matrix of the wall which do not occur acutely, but rather occur over time with chronic processes such as long-standing hypertension and aging.

Pressure must be measured relative to a reference value which is defined as zero. Most often the reference for zero pressure is atmospheric pressure because this is the pressure that surrounds the body. The pressure inside a vessel relative to the pressure outside a vessel gives the pressure that distends the wall of the vessel and is called transmural pressure. For example, if the outside atmospheric pressure is called zero and the pressure inside the aorta is 120/80 mmHg, the transmural pressure simply is 120/80 mmHg. However, if atmospheric pressure is 760 mmHg, the real pressure across the arterial wall relative to absolute zero pressure is 880/840 mmHg, but if this absolute value were to be used to determine if transmural pressure changed, one would have to first determine if atmospheric pressure had changed!

Tension across vessel walls often is calculated with use of the Laplace relationship and the value of pressure inside the vessel relative to atmospheric pressure. However, the Laplace relationship assumes that the wall is very thin relative to the radius of the structure, as is the case of a soap bubble [5]. Thus, although commonly used, this simplification is not valid for vascular structures and the full equation for the assessment of wall tension must be used with the pressure values relative to absolute zero pressure [6, 7]. When tension is calculated this way, the tension across the wall is a negative value in most vessels, which means that vessels are tending to explode rather than collapse, and wall tensions are actually more or less negative values.

### Kinetic energy

The second force determining arterial pressure is kinetic energy, which is due to the velocity of the flowing blood [8]. Kinetic energy is equal to the product of one half the mass (m) of the blood, which is the product of the volume and density of the blood, and the square of blood velocity (v):

$$ \mathrm{Kinetic}\ \mathrm{energy}=\left(1/2\ \mathrm{m}\times {\mathrm{v}}^2\right). $$

Velocity of flowing blood is in units of distance over time. The product of the velocity of blood and the cross-sectional area of a vessel gives flow of blood in units of volume per time. Kinetic energy contributes only about 3% of the total force at the peak of normal systolic pressure, but kinetic energy makes up a greater proportion of the pressure in large veins and pulmonary vessels because the velocity of blood is similar to that in the large arteries, whereas the elastic energy is much smaller.

**Fig. 2:**
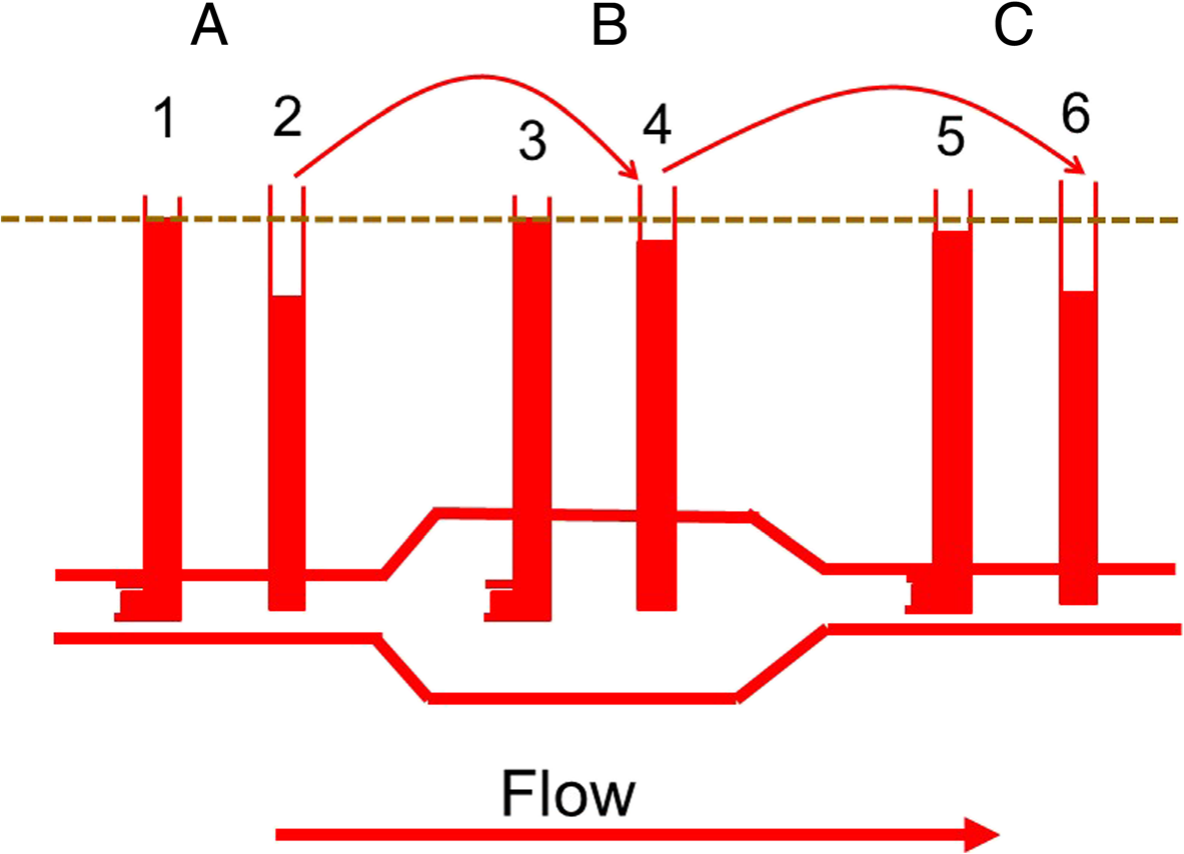
Pressure measurements in a vessel with an aneurysmal region. Pressures measured with fluid filled tubes facing the flow [1, 3, 5] measure elastic and kinetic energy, whereas tubes with the opening perpendicular to the flow just measure lateral pressure [2, 4, 6]. An assumption is that the energy loss due to resistance (*dashed line*) is minimal. In *A*, the tube facing the flow [1] shows a higher pressure than the tube measuring lateral pressure [2] because it includes kinetic energy. In *B* the vessel diameter is larger and velocity of flow is slower. The kinetic energy converts into elastic energy and the difference between tubes 3 and 4 is much smaller than between 1 and 2. In *C*, the tube narrows again so that kinetic energy increases and lateral energy decreases, which again increases the difference between 5 and 6

Kinetic energy can produce some confusing results, including blood seeming to flow from a lower to a higher pressure and the appearance that blood is flowing uphill! This occurs because flow is based on the total energy difference across a system, and not just the difference in elastic energy. Examples of this occur when sections of vessels either widen or narrow. Figure 2 shows an example of an aneurysmal dilatation of a vessel. Pressure is measured with a fluid filled catheter with the opening facing the oncoming flow, as is the practice with most arterial catheters, and another catheter that has an opening perpendicular to the flow (side pressure). Flow in L/min must be the same in each section of the vessel because what goes in must go out to maintain conservation of mass. However, in the region of the aneurysmal dilatation where the diameter is much larger, velocity is much slower because the cross-sectional area changes with the square of the radius. Energy cannot be created nor destroyed so the decrease in kinetic energy is converted into elastic energy. This increases the pressure on the wall of the dilated area and leads to further dilatation and a further increase in pressure on the already weakened wall until the wall stretches to a critical value and ruptures. In the vessel segment distal to the aneurysm, the velocity is again higher and elastic energy is converted back into kinetic energy so that it looks like blood is flowing from an area of lower pressure to an area of higher pressure when a catheter facing the flow is used.

Kinetic energy likely has a greater role in septic patients with high cardiac outputs, for the higher flow means that there is a greater kinetic component and, at the same time, elastic energy is decreased by the vasodilation. This will produce a difference between pressure measured with intravascular catheters facing the flow and the pressure measured with a non-invasive device which only measures the lateral elastic component of energy. Furthermore, the decreased lateral elastic force could alter myogenic responses whereas the increased velocity in small vessels will alter shear stress and the two could alter proper matching of flow to the tissue’s metabolic needs.

### Gravitational energy

**Fig. 3:**
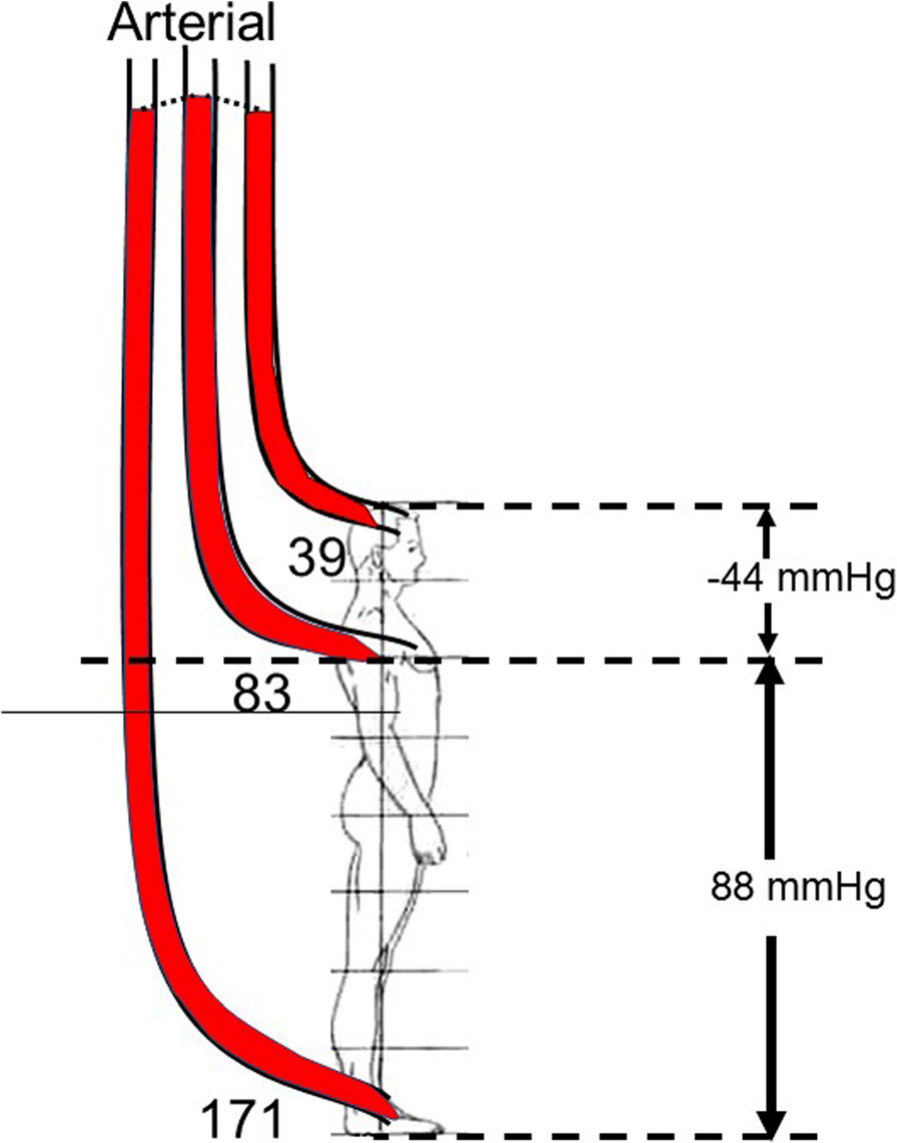
Gravitation effect on arterial pressures (adapted from [9]). The numbers on the *right* in mmHg refer to the gravitational potential energy related to the difference between the base of the measuring device relative to the mid-point of the right atrium (*dashed line*) assuming a 182-cm male. The loss of pressure due to resistance is assumed to be 5 mmHg. When the transducer is set at the level of the right atrium, the mean pressure is 83 mmHg. At the top of the head the pressure would only be 39 mmHg. If the transducer was at the level of the foot, the pressure would be a mean of 171 mmHg

The importance of the gravitational component of the energy for blood flow is important when pressure is measured with a fluid filled system. This is because the position of the transducer and the choice of the reference level have a large impact on the measured value and it is essential that the reference level is standardized. The gravitational effect on the body is very significant in the upright position. For example (Fig. 3), in a person who is 182 cm tall, and who has a systolic pressure of 110/70 mmHg and mean pressure of 83 mmHg measured at the level of the heart, the pressure measured with a transducer placed at the top of the head is only about 66/26 mmHg with a mean of 39 mmHg. On the other hand, if the transducer is placed at the level of the foot, the pressure would be 198/158 mmHg and a mean of 171 mmHg. It is worth noting that normal pressures for brain perfusion in the upright posture are much below clinically recommended targets but we do not need norepinephrine to walk around!

Gravitational energy is not a large factor for assessment of arterial pressure in the supine position, but it still is a significant factor for venous return because venous pressures are low, and the pressure difference between the region of systemic venous compliance and the right atrium is in the range of only 4 to 8 mmHg, which is about 6 to 11 cm of height. This can produce differences in venous return in the supine and prone positions and consequently in cardiac output [9].

## Determinants of arterial pressure

The main determinant of arterial pressure is the stretch of the walls of the arteries by the volume they contain. This volume increases in systole because inflow exceeds outflow and falls after the peak of ejection because outflow exceeds inflow. The outflow is dependent upon the resistance emptying the arterial tree and the elastance of the vessel walls. The product of the inverse of elastance (compliance) and the downstream resistance gives the time constant of emptying of the arterial vessels. The time constant is the time it takes to get to 63% of a new steady state after a step change in flow or pressure. Time constants are important in pulsatile systems because they set the amount of filling and emptying of aortic volume that can occur based on the cardiac frequency, the proportions of contraction and relaxation times during systole, and the diastole time.

### Resistances

Resistance to flow in a tube is given by Poiseuille’s law, which says that, in a tube with laminar flow, the resistance, which is the frictional loss of energy, is determined by the length of the tube, the viscosity of the blood, and the inverse of the radius of the tube raised to the fourth power [4]. Vessel radius is thus the dominant determinant of resistance and the only factor that can significantly change rapidly. The total resistance of tubes in series is determined by summing up all the individual resistances in the series; in contrast, the sum of parallel resistances is determined by:

1/Rtotal = 1/R1 + 1/R2 + 1/R3…1/Rn

**Fig. 4:**
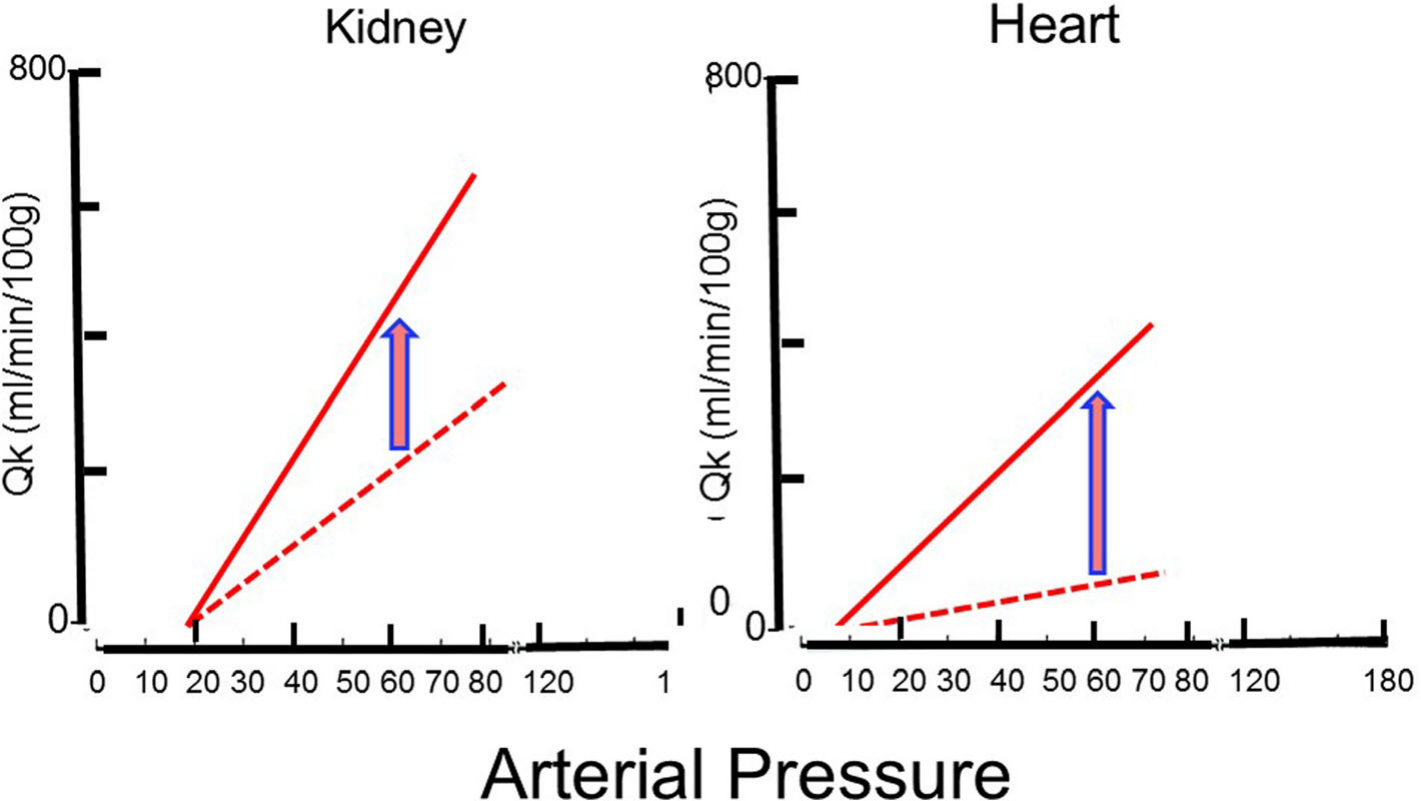
Flow vs pressure for kidney (*left*) and heart (*right*) based on data from hemorrhaged dogs [11]. The *dotted line* indicates baseline state and the *solid line* indicates maximal vasodilation with nitroprusside. The initial flow vs pressure line for the kidney is steep and is only a little steeper with vasodilation. The heart starts with a much flatter flow vs pressure line but can increase fivefold in the pressure range of 70–80 mmHg. Note that the peak conductance of flow to the heart is only mildly higher than the baseline conductance to the kidney

This is because the greater the number of parallel channels, the greater the overall cross-sectional area, and the greater the overall effective radius. Resistances vary among different vascular beds. Factors include the size of the vascular bed and the density of vessels. Because of their sizes, the splanchnic and muscle beds have overall low vascular resistances. However, when flows are related to the mass of tissue, muscle tissue has a high baseline resistance because the flow per mass is low. The importance of this is that the change in flow in different vascular beds with a fall in arterial pressure depends upon the slope of the pressure–flow line in that region [1]. The steeper the slope of the relationship, the greater the fall in flow for a given decrease in pressure. The kidney starts with a very steep pressure–flow relationship, whether assessed by mass or as a proportion of total body cardiac output, and it has a small capacity to dilate further [10] (Fig. 4).

The important factor in assessing the reserves of flow in a vascular region is the maximum slope of the regional pressure–flow line because this indicates the physical limit to flow at a given pressure (Fig. 4). Coronary blood flow can increase fivefold above the flow at a resting heart rate of 70 beats per minute. Thus, at low heart rates, the heart has very large blood flow reserves, which allow the heart to tolerate large decreases in arterial pressure. However, this is not true when there is a fixed coronary obstruction that limits the decrease in coronary resistance. On the other hand, the capacity to increase the slope of the pressure–flow relationship in the kidney is limited, which makes the kidney very sensitive to decreases in blood pressure.

### Critical closing pressure

Resistance to flow through a tube is calculated as the difference between the upstream and downstream pressures, divided by the flow between the two pressures. Accordingly, systemic vascular resistance typically is calculated as the difference between aortic mean pressure and the right atrial pressure, or central venous pressure, which usually are the same. This calculation assumes that the vascular system functions as a continuous tube, but this is not true. Most tissues have critical closing pressures at the level of the arterioles. These are also called vascular waterfalls or Starling resistors [11]. The presence of a critical closing pressure creates the same phenomena that exist in veins when the pressure inside a vessel is less than the pressure outside, but in arterioles flow limitation likely is created by the flow characteristics in small vessels without true collapse. When waterfall-like properties exist, the downstream pressure no longer effects flow, and arterial resistance should be calculated from mean arterial pressure to the critical closing pressure, and not to the right atrial pressure. Animal studies suggest that the average critical closing pressure for the whole circulation is around 30 mmHg [12] but the critical closing pressure differs among vascular beds [13]. For example, in resting skeletal muscle the critical closing pressure was estimated to be over 60 mmHg [14]. In the coronary circulation the critical closing pressure likely is in the range of 15 to 25 mmHg under baseline conditions [15]. Unfortunately, mean arterial critical closing pressure currently cannot be assessed in an intact person either for the whole body or in local regions.

When a critical closing pressure is present, use of the right atrial or central venous pressure as the value of downstream pressure for the vasculature produces an important error in the common assessment of vascular resistance. This is because the slope of the true flow versus pressure relationship, i.e., the inverse of resistance, is much steeper than that obtained with this standard calculation. Even worse, the error gets larger the lower the pressure or flow because the pressure below the critical closing pressure does not affect flow yet it takes up an increasingly greater proportion of the total pressure used for the calculation. This error makes it look like there is an increase in vascular resistance when flow decreases, which would make sense physiologically as being a defense against a fall in arterial pressure, but it occurs from the measurement error even if there is no actual vasoconstriction. This error makes it difficult to know if a drug such as milrinone improved cardiac output by its inotropic action or because it dilated vessels and reduced afterload. To truly know what happened, it is necessary to have two points on a pressure–flow line, but this cannot be readily obtained in human subjects, and for the matter, it is not easy to obtain in most animal studies. A useful point is that if the cardiac output rises with a rise or no change in arterial pressure, there was a true increase in cardiac function. The message is that resistance numbers are of little use and noting the relative change in blood flow and blood pressure is much more useful.

The arteriolar critical closing pressure is increased by a decrease in the carotid sinus pressure and alpha-adrenergic agonists [16, 17]. It is decreased by increased arterial pressure through the myogenic response [18] and by calcium channel blockers [19]. It also decreases with reactive hyperemia and exercise-induced hyperemia [14, 20], indicating that it also responds to local metabolic activity.

### Cardiac-aortic coupling

**Fig. 5:**
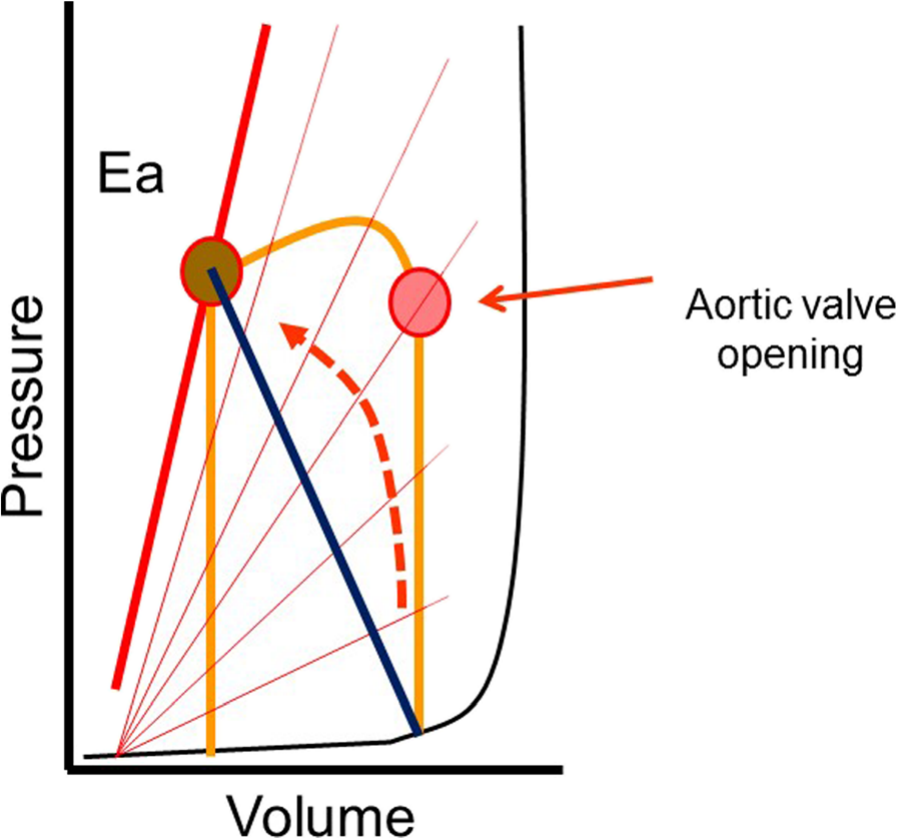
Pressure versus volume relationship of the left ventricle. The series of lines with increasing slopes indicate the time-varying elastance of the aorta as described by Sagawa and colleagues [22]. Note that aortic valve opening occurs much before peak aortic elastance, peak left ventricular pressure, and accordingly peak aortic pressure

The main determinant of the stroke volume by the ejecting heart is the pressure at which the aortic valve opens, because this is the pressure at which heart muscle begins to shorten with a quasi-isotonic contraction (Fig. 5). When the aortic valve opens, the left ventricle is not yet at peak systolic elastance, and ejection continues until maximal left ventricular elastance is reached [21, 22]. Maximum ventricular elastance, i.e., the slope of the end-systolic pressure–volume line, is only a property of the heart and it is not a function of the load on the heart. The slope of this relationship is the same whether the heart contracts isometrically or isotonically [21]. The diastolic pressure at which the aortic valve opens is a function of the volume that is still in the aorta at the end of diastole. That volume is determined by a composite of factors: the amount of volume that was put into the aorta during the previous systole, the time allowed for the volume to empty, which is dependent upon the length of diastole, the downstream arterial resistance, the critical closing pressures in small arteries or arterioles, and aortic elastance. The resistance and compliance (inverse of elastance) of the aortic wall determine the time constant of arterial emptying and the volume left in the aorta at the end of each cycle. An increase in true aortic elastance (i.e., the shape and position of the whole curve; Fig. 1) is important because it is a determinant of the diastolic pressure at which the aortic valve opens, the shape of the pulse pressure, and the speed of the forward and backward pressure waves in the aorta [23, 24]. Ultimately, the final value of arterial pressure is set by the strong regulatory mechanisms that ensure that cardiac output and the return of blood to the heart match metabolic needs and as adjustments in vascular resistance and regional critical closing pressures to maintain a constant arterial pressure. This means that arterial pressure should not be considered in isolation.

### Dynamic elastance

Dynamic elastance has recently become popular. It is argued that it can be a useful measure for assessing the coupling of the heart and circulation [25–28]. It is derived from concepts introduced by Sunagawa and co-workers [29, 30], who attempted to derive an equation that relates stroke volume to the mechanical properties of the ventricle and vascular system. Their equations predicted stroke volume based on derived aortic and ventricular elastances. Unappreciated requirements were that ventricular diastolic pressure was considered to be on the flatter part of the ventricular diastolic filling curve, and that heart rate was constant, neither of which can be assured in the intact circulation. When these assumptions are true, the prediction of stroke volume from the formula essentially represented the ascending portion of a cardiac function curve with a constant heart rate, contractility, and afterload.

The term dynamic elastance currently used by investigators [26–28, 31] is based on the ratio of respiratory variation in pulse pressure that occurs with each positive pressure breath as a percentage of the mean pressure divided by the corresponding change in stroke volume as a percentage of the mean change during the breath. This makes for a very complex measure. True elastance can only be assessed in a static state by increasing or decreasing volume in an elastic structure by a known amount with no flow and then observing the change in pressure. Elastance is also different in the thoracic and abdominal aorta and in the different large vessels [32]. The total elastance is determined by the sum of the elastances of all the arterial vascular segments.

When flow is present, especially pulsatile flow, there are also resistance and kinetic components to this dynamic measure. A further problem is the curvilinear shape of the aortic volume–pressure relationship. Because of this shape, the change in pressure with a change in volume is greater at higher initial volumes because the volume is moving up the steeper part of the relationship, but the actual shape of the relationship itself is constant over short periods of time. It can become stiffer over time with increases in age and hypertension (Fig. 1). The clinically obtained “dynamic” elastance is not a static measurement and is dominated by changes in arterial resistance [29], the critical closing pressure, and, to some extent, the position on the arterial volume–pressure curve. Since the dynamic elastance term uses cyclic respiratory challenges to produce changes in pulse pressure and stroke volume, the changes likely are occurring mainly through the changes in return of blood to the right heart and to some extent by changes in loading of the right heart with lung inflation. This means that this measurement is affected by changes in blood volume, the size of the change in pleural pressure, and the change in transpulmonary pressure. Heart rate too is a factor because the length of diastole is a determinant of the volume that remains in the aorta at the end of diastole and thus a determinant of where the arterial volume is on the elastance curve [33]. It also is affected by the emptying of the pulmonary venous reserves during the respiratory cycle [34]. The respiratory rate and length of inspiration and expiration add other factors. It is thus not surprising that dynamic elastance does not always act as expected [31] and at best may reflect general patterns. It is likely preferable to just examine the change in stroke volume, cardiac output, and blood pressure that were used to derive the measurement to interpret the response to a therapy.

## Regulation of arterial pressure

Arterial pressures in all mammals from mice to humans is in the same range and blood pressure is one of the body’s most tightly regulated variables. The feedback control is remarkable. A young male exercising to near maximum aerobic capacity can increase cardiac output fivefold or more but mean arterial pressure does not change or even slightly decreases. For this to occur, arterial resistance must decrease by more than 80%. This tight regulation of arterial pressure occurs primarily through baroreceptor regulation, but regional myogenic mechanisms and metabolic activity also contribute to local autoregulation. Flow-mediated adjustments also occur, by which increased flow produces a decrease in downstream vascular resistance by the release of nitric oxide from the endothelium [35].

The tight control of blood pressure raises the physiological question as to why this evolved and why is arterial pressure much higher in mammals and birds than in all other species? The high arterial pressure is not necessary for baseline flow because the full cardiac output goes from the right to left heart through the lungs with a mean pressure of only 10 to 15 mmHg. Pulmonary arterial pressure remains low at peak exercise even with a fivefold increase in cardiac output. There are two main advantages for our high systemic arterial pressures. First, by keeping arterial pressure relatively constant, regional flows can change by altering regional arterial resistances according to regional needs for flow, without a change in aortic pressure. This works much like opening taps in your house, which allows a common pressure head to allow water to flow into the sink, bathtub, or toilet. The alternative way to increase flow to a region, such as the working muscle, would be to increase blood pressure by increasing the resistance in every vascular region except for the one that needs more flow. This obviously is a much more complex process than simply dilating one region and would have major consequences for regions that did not need more flow. It also would mean that the initial low arterial pressure would have to increase when there is a regional need for more flow, and this would increase the strain on the heart. This brings up a second advantage for having a high baseline arterial pressure. Because mean aortic pressure changes little with changes in regional flows or cardiac output, the load on the heart is relatively constant. This is important because the heart tolerates pressure loads much less well than volume loads (i.e., volume ejected) so that by having a relatively constant pressure, the load on the heart is relatively constant.

### Autoregulation

It often is argued that ideal targets for perfusion pressure should be in the range of normal autoregulated blood flow [13]. This is defined as the pressure range in which flow does not change with increases or deceases in pressure [36]. Maintenance of flow with changes in arterial pressure can occur through four general mechanisms that regulate vascular resistance and regional critical closing pressures: neuro-humeral, myogenic, metabolic, and flow-mediated processes. However, the range of autoregulated flow, the mechanisms that regulate autoregulation in a tissue, the effects of drugs, the effects of disease, and the effects of central nervous activity differ among vascular beds and cannot be generalized.

Let us start with what are the dominant controlled variables in the whole circulation. As already stated, we are pressure-regulated beings, meaning that maintenance of a constant blood pressure is a priority for the body. Blood pressure is approximated by flow (cardiac output) and the systemic vascular resistance. Cardiac output is determined by metabolic needs of tissues, which can be quantified by their consumption of oxygen. Considering that control of arterial pressure is a priority for the body, and that cardiac output is strongly related to metabolic needs, it can be appreciated that changes in systemic vascular resistance dominate the normal regulation of arterial pressure. Regulation of systemic vascular resistance first occurs through neural mechanisms that provide afferent feedback to the medullary cardio-inhibitory and cardio-stimulatory regions that regulate vascular tone by sending efferent signals through parasympathetic and sympathetic pathways, and to some extent through humoral signals, to maintain the centrally set pressure.

The relationship of blood flow to metabolic need for the whole body is dominated by tissues that can greatly increase their oxygen needs, which are skeletal and cardiac muscle. In these tissues, as is the case for the whole body, there is a linear relationship between blood flow and oxygen consumption, indicating that the primary regulator of blood flow is metabolic activity. This strong metabolic coupling can override neural-mediated vasoconstriction. Metabolic activity likely plays an important role in the brain, too; however, the limited space in the skull means that increased volume and pressure must be controlled, likely by the myogenic process.

The two dominantly controlled cardiovascular variables, systemic arterial blood pressure and cardiac output relative to metabolic need, can be in conflict. A fall in arterial pressure with a normal cardiac output requires an increase in systemic vascular resistance to restore arterial pressure, but the rise in arterial resistance increases the load on the left ventricle, which could lead to a decrease in cardiac output. The hypotension would be fixed, but tissue perfusion would not. If the increase in vasoconstriction also increases venous resistance, cardiac output would fall even more [37]. If the fall in arterial pressure occurs because of a decrease in cardiac output, an increase in arterial resistance in all vascular beds will restore blood pressure, but not regional organ blood flows. The hope when a pure vasoconstrictor drug is used is that local metabolic activity will override the constricting effect of the drug in critical vascular beds such as the brain and heart so that these regions will receive a greater proportion of the available flow. How much this occurs likely depends upon the ability of these regions to modify the generalized vasoconstriction through their local signals, and likely also is affected by the receptor density for the vasoconstricting drug. Very high doses may just constrict all regions non-discriminately. The clinically important point is that if tissue perfusion is low, a treatment must increase cardiac output without a change in arterial pressure and not overwhelm regional mechanisms that match flow to tissue needs. This type of strategy requires some measure of blood flow or indirect measures of tissue perfusion such as lactate and central venous saturation as well as clinical indicators such as wakefulness, skin temperature and color, and urine output when the kidneys are working.

Baroreceptor-induced vasoconstriction is greater in peripheral vascular beds, which are primarily muscle tissue, than in the splanchnic bed [38, 39]. This shifts the distribution of blood flow to the splanchnic bed. By itself this would result in a decrease in cardiac output and a further decrease in blood pressure because volume accumulates in the very compliant splanchnic vasculature [40, 41]. However, the effect of this redistribution is compensated for by a decrease in the capacitance in the splanchnic bed (recruitment of unstressed into stressed volume) and a decrease in splanchnic venous resistance at the same time as the arterial resistance to the splanchnic bed increases [39]. It is likely that infused vasoconstrictors also affect the peripheral vasculature more than the splanchnic bed, but at higher doses the difference may no longer be active and these drugs may then alter the normal distribution of resistances. If the vasoconstrictor cannot recruit more unstressed volume because there are insufficient reserves, and constricts venous resistance, cardiac output and tissue perfusion will fall. Tissues need flow and not pressure unless they can selectively dilate. This is what happens in most cases when phenylephrine is given; arterial pressure rises but cardiac output falls [37, 42]. In contrast, norepinephrine in moderate doses does not increase venous resistance and also produces a moderate increase in cardiac function [43]. Again, monitoring perfusion or cardiac output can be helpful to know what is happening.

The fourth factor regulating local blood flow is flow-mediated dilatation. This provides a feed-forward process and decreases the downstream resistance when flow increases [44]. It primarily is mediated by release of nitric oxide (NO) through the effect of shear stress on vascular endothelial cells [45]. This mechanism would spiral out of control if something else does not happen because the decrease in downstream resistance would result in more flow, more release of NO, greater flow, and so on. It is typical of nature to drive with her foot on the gas and brake at the same time; consider simultaneous parasympathetic and sympathetic nerve activities and the cardio-inhibitory and cardio-stimulatory centers in the brain. In this case the brakes are the local myogenic and central neuro-humeral mechanisms, as well as local metabolic needs. The advantage to such a process is that it allows rapid adaptation to increased needs for flow and fine tuning of the matching of flow to local metabolic activity. Flow-mediated dilatation is lost when the endothelium is damaged in vascular disease and contributes to further vascular damage.

Returning to the question of the usefulness of targeting the autoregulatory range, I would argue that what really counts is avoiding the lower autoregulatory range in which flow falls when arterial pressure falls and, even more so, when this is combined with a decrease in oxygen consumption, because dilatation and oxygen extraction are maximal. When this limit is reached, the only treatments that can help tissue perfusion are an increase in cardiac output or constriction of some other region, but constriction of these other regions would compromise their function. This means that organs cannot be considered in isolation and the reserves of the whole system need to be considered.

## Conclusions

Mean arterial pressure is determined by cardiac output, systemic vascular resistance, and a critical closing pressure at the level of the arterioles. Each of these factors is controlled by mechanisms that work at the level of the whole organism, but also interact with important local regulatory mechanisms. Arterial pulse pressure brings in another set of variables which are related to the elastance of the aortic wall, the volume of blood in the aorta, the cardiac frequency, and the proportion of time in systole and diastole. Because of the complexities of these interactions, it is not possible to make simple predictions of the response to vasopressor therapies. This becomes even more complicated when pathologies are added that alter the potential of vessels to respond, or because there are fixed obstructions to flow. Only empiric studies can determine the best approach for the management of hypotension and hypoperfusion. Finally, it must be remembered that what counts for tissues is blood flow and not the arterial pressure and, even more importantly, the matching of flow to metabolic needs. The body does this masterfully through multiple counteracting control mechanisms. It is very unlikely that a single therapeutic agent can match the naturally occurring well-orchestrated control mechanisms.

## Abbreviations

cm: Centimeter
L: Liter
m: Mass
mmHg: Millimeter of mercury
R_n_: Segment resistance
R_Total_: Total resistance
v: Velocity (L/sec)
